# Phisiological Antagonism between Medicines and Diseases

**Published:** 1896-03

**Authors:** 


### Physiological Antagonisms between Medicines and Diseases.

Read before the Isaac Knapp Dental Coteries of Ft. Wayne, Indiana.

To the Isaac Knapp Dental Coterie of Ft. Wayne, Indiana. The pleasure which I feel at this time originates from two sources— First, that dentistry is sufficiently advanced that members are able to discuss such a theme. Second, that congenial natures can converse with each other when many miles apart.

I wish to call attention to the histological structure of a tooth and surrounding tissues—not all of the textures, but the capillaries. From a physiological view, the capillaries commence at a point where blood is brought near enough to the tissues to enable them to separate the elements necessary to their regeneration, and to give up the products of decay. (*Flint.*)

The capillaries are those vessels which have but a single tunic, called the endothelial coat, and they are $\frac{1}{3000}$ of an inch in diameter. The endothelial coat is composed of cell plates with an oval nucleus and is elastic. The capillary is so small that blood corpuscles move in a single row, and even become deformed in passing through the smallest capillaries. (*Klein.*)

### THE PHENOMENA OF CAPILLARY CIRCULATION.

The corpuscles occupy the central portion, and the plasma forms a clear layer at the sides and seems almost motionless. The blood in the capillaries has no definite direction, at one moment they (corpuscles) move in one direction, and the next moment an opposite current may be seen in the same capillary. The capillaries do not receive the impulse of the heart, and the velocity is generally estimated at one inch in ninety seconds.

### CAUSE OF CAPILLARY CIRCULATION

First: The heart's contraction. Second: The reaction of the elastic tissues surrounding the blood current. (*Dalton.*)

Please remember that the vessels which I have described, the very peculiar movement of the blood through them, and the forces which propel the vital fluid, *is the means* by which the blood gives to the pulp, cementum, peridental membrane, alveolar wall and gum tissues the elements which sustain their vital condition.

Now, the point which I wish to make is this: All that is stated above is a condition which we can call healthy, normal, or physiological, and any deviation is abnormal, *i. e.*, disease, or a pathological state. The distension of the capillaries, the determination of blood to fill them, the movement of corpuscles so fast that they can hardly be distinguished, the stagnation of the current, the forcing of corpuscles through the vessel-wall, etc. —these deviations from the normal, establish a condition which has four striking characteristics, viz: redness, heat, swelling and pain. This is called inflammation; but to confine ourselves to the term used in the subject, we will call it disease. Now, what will cause this disease? I will say that anything *foreign* to the process which we described as physiological will establish the pathological or diseased condition. The air, food and saliva are foreign to the dental pulp, and when brought in contact, the diseased pulp begins an inflammatory campaign. A nerve broach, the gas from a decomposed pulp, or anything forced into the apical space is *foreign* to the tissue and will be a source of disease as long as it remains.

Now, the question is, how does medicine antagonize this inflammatory condition? Let us call ice, cold water, heat by means of a poultice or hot pillow, creasote, arsenic, nitrate of silver, capsicum or alcohol medicines. We apply these agents externally. How do they reduce or subdue the condition which we style disease?

We have all noticed that a person holds the face to the fire or rests the head on a warm pillow or applies a poultice to relieve the pain.

Can there be any virtue in these applications? Let us note now that in the above examples, *heat is the virtuous agent* and that *it exerts its influence on the circulation of the cheek* and may antagonize the disease in an exposed pulp or a diseased peridental membrane. We might dismiss the case by saying that this is *counter-irritation*, that is, the heat induces the blood to collect in the cheek and thereby reduce the engorged condition in or about the tooth. This is a true explanation, but it is superficial, inasmuch as our subject would ask us to tell *how heat antagonizes disease.* We may make an explanation which sounds more scientific, but it tells but little more than the counter-irritation or counter-congestion theory. When heat is applied to the surface of the cheek it paralyzes the nerve filaments which supply the muscular tissue of the vessels before they merge into capillaries—this paralysis overcomes the contractile energy of the muscular tissue—therefore the vessel must expand. So the expansion being followed by a determination of blood to the cheek will reduce the volume of blood in the part which is inflamed. But shall we call this a physiological antagonism between heat and inflammation? Do we not set up a pathological condition in the circulation of the cheek in order to reduce the energy of a pathological condition in the tooth?

A capsicum pad placed on the gum over an inflamed root acts in the same way and admits of the same explanation.

*Any irritant*, as carbolic acid or nitrate of silver, applied a short distance from the seat of disease will act in the same way if there is not enough used to induce an irritation which is so aggravating as to end in a collapse of the vital processes, *i. e.*, death of the part.

When a person comes in with a raging tooth, the pain is the principal symptom of disease, and if the nerve is exposed we usually proceed to treat the case with creosote, arsenic or some powerful irritant. Here we have such an antagonism between the medicine and disease that not even a pathological state can continue—here the safety of the case depends upon murder, but we can never be charged with incompetency. If nature had supplied the dental pulp with lymphatics—if it ever becomes possible for us to relieve the pulp from the influence of foreign substances—such as pus, serum, extravasated blood, particles of decay, bacteria, pressure from fillings, etc., it will then be proper to discontinue the wholesale slaughter of life within the teeth. The only thing we can teach is *come in time.*

But our subject requires us to explain the antagonism between the medicine we use and the disease.

When an irritant is applied to the tissue, the result is contraction of the arteriole and diminution of velocity in the circulation. If the irritant be powerful as arsenic the contraction is momentary—this is followed by dilatation and increased velocity. Soon oscillation takes place which is followed by complete stagnation. In this condition there is no nutrition and the result is death; the antagonism is so violent that, not only disease, but all life goes down in the conflict.

